# Non-Uniform Embedding Scheme and Low-Dimensional Approximation Methods for Causality Detection

**DOI:** 10.3390/e22070745

**Published:** 2020-07-06

**Authors:** Angeliki Papana

**Affiliations:** Department of Economics, University of Macedonia, 54006 Thessaloniki, Greece; angeliki.papana@gmail.com or apapana@uom.edu.gr

**Keywords:** multivariate time series, connectivity, Granger causality, non-uniform embedding, low-dimensional approximation of CMI, financial network

## Abstract

Information causality measures have proven to be very effective in uncovering the connectivity patterns of multivariate systems. The non-uniform embedding (NUE) scheme has been developed to address the “curse of dimensionality”, since the estimation relies on high-dimensional conditional mutual information (CMI) terms. Although the NUE scheme is a dimension reduction technique, the estimation of high-dimensional CMIs is still required. A possible solution is the utilization of low-dimensional approximation (LA) methods for the computation of CMIs. In this study, we aim to provide useful insights regarding the effectiveness of causality measures that rely on NUE and/or on LA methods. In a comparative study, three causality detection methods are evaluated, namely partial transfer entropy (PTE) defined using uniform embedding, PTE using the NUE scheme (PTENUE), and PTE utilizing both NUE and an LA method (LATE). Results from simulations on well known coupled systems suggest the superiority of PTENUE over the other two measures in identifying the true causal effects, having also the least computational cost. The effectiveness of PTENUE is also demonstrated in a real application, where insights are presented regarding the leading forces in financial data.

## 1. Introduction

Causality is the relationship between cause and effect. In other words, there is a causal relationship between two situations when it is certain that the second one arose due to the first one. The causal link is not mentioned exclusively in the relationship between two events or situations alone, but a causal chain may exist between causes and effects. The key component of causality is the succession of cause and effect. Reinchenbach [1] was the first to point out that the hypothesis of causality in real phenomena should be questioned and not taken a priori as granted. The perspective that the future is indeterminate and the connection between indeterminism and a dynamic view of the world are discussed in this work. Reichenbach [2] also postulated the principle of common cause, i.e., the dependence of two variables can be explained by at least one of the following cases: there is a unidirectional or bidirectional causation between the variables, or there exists a common cause of the two variables.

Identifying the connectivity pattern in complex multivariate systems is an issue that has seen enormous advances recent years. A variety of methods has been developed and compared, leading sometimes to contradictory conclusions [3,4,5,6,7,8]. Understanding the pros and cons, the estimation accuracy and computational cost of each causal discovery method are of great importance in exploiting the optimal one for experimental and empirical tasks.

A traditional way to discover causal relations is to use interventions or randomized experiments. If this is not possible, causal information is revealed by analyzing the statistical properties of purely observational data [9]. Probabilistic causation characterizes the relationship between cause and effect based on probability theory. The key idea is that causes raise the probabilities of their effects. Probabilistic theories of causality rely on the basic intuitions in Good [10] and Suppes [11], pointing out the temporal priority of the cause and the statistical relevance requirement, which serves as a measure of the strength of a causal chain.

The benchmark work of Spirtes et al. [12] utilized Bayesian network models for causal discovery. A review of causal discovery methods based on graphical models can be found in [13]. Recently, additional causal discovery methods have been proposed in the literature. Algorithmic information dynamics is an algorithmic probabilistic framework for causal discovery and causal analysis, which does not rely on graphical models or empirical estimation of mass probability distributions, as traditional probabilistic methods do [14,15,16].

Granger’s concept of causality is based on Wiener’s study [17], according to which, if the forecast of a time series is improved by incorporating the information of a second one, then the latter exerts a causal effect on the first. Granger formalized this idea in the context of linear regression [18]. A variable *X* Granger causes *Y*, if all the recent and previous information on *X* values helps in the better prediction of the *Y* values.

The notion of Granger causality has been very popular the last few decades, and various extensions of its original form have been introduced and applied in an ensemble of scientific fields. However, such approaches have also been intensively criticized [19,20]. Temporal precedence alone is not a sufficient condition for establishing directional relationships. One key problem is that such methods lead to spurious causal influences by the omission of relevant variables. In a review paper on the concept of Granger causality for causal inference from time series data, Eichler [21] concluded that: “Causal inference based on Granger causality is indeed legitimate, but in many cases provides only sparse identification of true causal relationships, that is, for most links among the variables it cannot be determined whether the link is truly causal or not. Correct learning accumulates knowledge obtained from the large variety of possible submodels. This imposes feasibility constraints in the size of the networks that can be practically analyzed. Any analysis claiming full identification of the causal structure either must be based on very strong assumptions or prior information or—on closer inspection—turns out to be unwarranted.”

Recently, methods of nonlinear time series analysis [22,23] and complex networks [24,25] have been jointly examined. In a mathematical formulation, the set of observed time series constitutes the observed variables of a complex system of which the interdependence structure is to be investigated. Further, graph theory [26] offers a tool to visualize the structure of a complex system as a complex network, where the nodes are the observed variables and the connections can be formed utilizing an interdependence measure.

Pairwise causality measures indicate the direct and indirect causal effects between two time series. In [27], the authors pointed out some pitfalls in applying pairwise measures in the case of mutually dependent time series, while noting that commonly used pairwise measures often lead to erroneous results. On the other hand, conditional (or direct) causality measures exploit all the available information of the observed data to infer the connectivity. Granger [28] addressed the problem of missing information and stated that a test for causality is impossible unless the set of interacting channels is complete.

The standard Granger causality test [18] quantifies the directed interrelationships based on vector auto-regressive (VAR) models for prediction. Its nonlinear analogue from information theory is transfer entropy (TE) [29]. Partial transfer entropy (PTE) extends the pairwise TE and indicates only direct causal influences. The computation of PTE relies on uniform state space reconstruction. Different estimators of PTE have been introduced, with *k*-nearest neighbors (KNN) being one of the most effective ones [30].

In the framework of information theory, the computation of conditional causality measures requires the estimation of marginal and joint probability densities. The prediction of the future of the response variable is done using entropy terms. In the case of a large number of observed variables, the problem of estimating high-dimensional densities arises, which is commonly called the “curse of dimensionality” and affects the reliability of causal inference.

The non-uniform embedding (NUE) scheme has been developed to address this problem along with the problem of irrelevance and redundancy. It was originally introduced for the pairwise case [31] and later expanded to the conditioned one [32,33]. This method progressively builds an optimal mixed embedding vector allowing for different variables and delays using a conditional mutual information (CMI) criterion. Information causality measures exploiting the NUE scheme have been examined on empirical and real datasets with very promising results [33,34].

Estimating CMI in multivariate systems requires the computation of high-dimensional joint probability distributions. To circumvent this hurdle, a low-dimensional approximation (LA) methodology has been proposed that helps compute CMI as the sum of mutual information quantities of lower dimensionality. The LA methodology has been combined with the NUE scheme for unraveling causality problems; however, the empirical findings do not seem to be clear regarding the effectiveness of such measures [35,36].

In this paper, we examine the performance of conditional information causality measures in correctly identifying the connectivity network of coupled systems and demonstrate the necessity of using dimension reduction techniques. We shed light on the importance of the NUE scheme and also demonstrate the combined effectiveness of the NUE scheme with an LA method. In particular, we compare partial transfer entropy (PTE) defined on the standard uniform embedding scheme, with two dimension reduction measures, PTE utilizing the NUE scheme (PTENUE) [34] and PTE based on the NUE scheme and an LA algorithm (LATE) [35,36]. The three causality measures are evaluated on synthetic time series with different characteristics, focusing on discrete systems. Numerical outcomes demonstrate the superiority of PTENUE, in terms of its strength and power, but also regarding its computational cost. Novel results are presented, emphasizing high-dimensional simulation systems. The superiority of PTENUE over the other two measures is manifested with a financial application, where the goal is to identify the leading forces among the examined variables.

In Section 2, the three causality measures, namely PTE, PTENUE, and LATE, are reviewed. The simulation analysis for the evaluation of the causality measures is presented in Section 3. Then, Section 4 highlights the efficiency of PTENUE on financial data. Finally, the findings are summarized in Section 5.

## 2. Methodology

Without loss of generality, let us consider the multivariate dataset in *K* variables, where *X* is the driving variable, *Y* is the response variable, and there are K−2 confounding variables, collectively denoted by $Z=X_{1},\ldots ,X_{K-2}$. The future of the response variable, $y_{t+1}$, is predicted for one step ahead.

### 2.1. Partial Transfer Entropy

Partial transfer entropy (PTE) is a causality measure from information theory that defines Granger causality based on entropy instead of VARs. It is model free and indicates both linear and nonlinear causal effects. The computation of PTE involves the formulation of uniformly spaced embedding vectors from each variable, e.g., for *X*, the corresponding embedding vector is $x_{t}={\left[x_{t},x_{t-\tau },\ldots ,x_{t-(m-1)\tau }\right]}^{\prime }$, where *m* is the embedding dimension and τ is the time lag. PTE accounts for the direct coupling of *X* to *Y* conditioning on the remaining variables of the multivariate system [30,37,38]:

(1) $$\begin{array}{cc} {PTE}_{X\to Y|Z} & =I(y_{t+1};x_{t}|y_{t},z_{t})\\ & =H\left(x_{t},y_{t},z_{t}\right)-H\left(y_{t+1},x_{t},y_{t},z_{t}\right)+H\left(y_{t+1},y_{t},z_{t}\right)-H\left(y_{t},z_{t}\right), \end{array}$$

where I(.|.) represents the conditional mutual information and H(.) Shannon entropy. For a discrete variable *X*, Shannon entropy is defined as $H\left(X\right)=-\sum p\left(x_{i}\right)\log p\left(x_{i}\right)$, where $p(x_{i})$ is the probability mass function of the outcome $x_{i}$, typically estimated by the relative frequency of $x_{i}$.

Entropy estimation can be performed based on random variable discretization by partitioning [39] or by estimating the probability mass according to the pdf [40,41]. For the estimation of the probability densities, the *k*-nearest neighbors’ estimator (KNN) is used [42]. The KNN estimator uses the distances between the reconstructed state space vectors to estimate the joint and marginal densities. For each reference point, viewed in the largest state space, the distance length ϵ is defined as the distance to the $k^{\mathrm{th}}$ nearest neighbor. Then, densities, at projected subspaces, are locally formed by the number of points within ϵ from each reference point. The KNN estimator has been shown to be stable, not significantly affected by the choice of *k*, and specifically effective for high-dimensional data [30,31,42,43].

PTE requires a significance test for the non-causality null hypothesis (H_0_). Surrogate time series are generated by randomly time-shifting the values of the driving time series, while the other series remain unchanged [44]. The time-shifted surrogates preserve the dynamics of the original time series (such as the histogram and autocorrelations), while the couplings between the driving variable *X* and the response *Y* are destroyed. They are formed by cyclically time-shifting the components of the driving time series $\left\{x_{1},\ldots ,x_{n}\right\}$: an integer *d* is randomly chosen, and the *d* first values of the time series are moved to the end, giving the time-shifted surrogate time series $\left\{x_{t}^{S}\right\}=\left\{x_{d+1},\ldots ,x_{n},x_{1},\ldots ,x_{d}\right\}$. The random number *d* is randomly drawn from the discrete uniform distribution in the range [0.05n,0.95n] in order to maintain disruption of the time order of the original time series even in the presence of strong autocorrelations. PTE is then estimated from 100 realizations per simulation system and time series length. For each realization, one-hundred surrogate time series are generated using the described time-shifting scheme. Let us denote $q_{0}$ the original PTE value from one realization of a system and $q_{1},q_{2},\ldots ,q_{100}$ the PTE values from the surrogate time series. The rejection of H_0_ is decided by the rank ordering of the estimated PTE values. If $r_{0}$ is the rank of $q_{0}$ when ranking the list $q_{0},q_{1},\ldots ,q_{100}$ in ascending order, then the *p*-value of the one-sided test is $1-\left(r_{0}-0.326\right)/\left(M+1+0.348\right)$, by applying the correction in [45]. The significance level is set equal to α=0.05.

The MATLAB code for the computation of partial transfer entropy using the KNN estimator has been uploaded on the GitHub (https://github.com/angelikipapana/PTE-variants).

### 2.2. Partial Transfer Entropy on Non-Uniform Embedding

The estimation procedure of PTENUE is based on the non-uniform embedding (NUE) scheme. In order to best explain the future of the response variable *Y*, a mixed embedding vector $w_{t}=\left[w_{t}^{X},w_{t}^{Y},w_{t}^{Z}\right]$ is formed, where lagged terms from all observed variables are chosen based on a conditional mutual information (CMI) criterion. Therefore, the lagged terms entering $w_{t}$ are not necessarily uniform over time, and dimension reduction is accomplished.

For a predefined maximum lag $L_{max}$, the ensemble of lagged terms for forming the mixed embedding vector $w_{t}$ is $B=\{x_{t},x_{t-1},\ldots ,x_{t-L_{max}},y_{t},y_{t-1},\ldots ,y_{t-L_{max}},z_{1t},\ldots ,z_{K(t-L_{max})}\;$. To formulate $w_{t}$, start with an empty vector. At each step *j*, form a new vector $w_{t}^{j}$ by adding a new component $w_{t}^{j}$ (lagged term from any observed variable) that gives the most information about the future of the response variable $y_{t+1}$, conditioning on the mixed embedding vector of the previous step $w_{t}^{j-1}$ (CMI criterion):

(2) $$\begin{array}{c} w_{t}^{j}={argmax}_{w_{t}^{j}}I\left(y_{t+1};w_{t}^{j}|w_{t}^{j-1}\right) \end{array}$$

The stopping criterion relies on randomization of the driving variable. The decision for a significant CMI is made by comparing the CMI of the original data with the 1−α percentile of the surrogate CMI values. PTENUE measures the direct effect of *X* on *Y* in the presence of the “appropriate” past terms of the remaining variables:

(3) $$\begin{array}{ccc} {PTENUE}_{X\to Y|Z} & = & I(y_{t+1};w_{t}^{X}|w_{t}). \end{array}$$

PTENUE was computed using the ITStoolbox (http://www.lucafaes.net/its.html). The free parameters for the computation of PTENUE were set as suggested in the ITS toolbox, i.e., the significance level for the stopping criterion was set to be α=0.01.

### 2.3. Low-dimensional Approximation of Transfer Entropy

The low-dimensional approximation (LA) methodology has been examined in the framework of feature selection [46,47,48]. Feature selection techniques aim to reduce the number of features, in order to resolve over-fitting problems and increase the computational speed for learning [49]. Model-independent filter procedures for feature selection are classifier agnostic, simple, and computationally efficient. The conditional mutual information (CMI) has been considered as a criterion for feature selection, e.g., by identifying the best subset of features that together have the highest CMI with the class variable.

Exploiting the LA methodology and the non-uniform embedding (NUE) scheme, we end up with another variant of partial transfer entropy (PTE), denoted as LATE [35]. The estimation procedure of LATE is similar to PTENUE’s; however, the selection of the lagged terms that form the mixed embedding vector is based on the sum of mutual information terms and low-dimensional CMIs instead of considering a high-dimensional CMI. Suppose that the set of conditioning vectors we have step-wisely built up to step j−1 is *V*. Then, the selection of the term $w_{t}^{j}$ in step *j* is extracted on the basis of the LA scheme as:

(4) $$\begin{array}{c} w_{t}^{j}={argmax}_{w_{t}^{j}\in B\;V}(I\left(y_{t+1};w_{t}^{j}\right)-2|B|\sum_{w_{t}^{i}\in B}I\left(w_{t}^{j};w_{t}^{i}\right)-2|B|\sum_{w_{t}^{i}\in B}I\left(w_{t}^{j};w_{t}^{i}|y_{t+1}\right)) \end{array}$$

After defining the mixed embedding vector $w_{t}$, LATE is expressed as:

(5) $$\begin{array}{ccc} {LATE}_{X\to Y|Z} & = & I(y_{t+1};w_{t}^{X}|w_{t}). \end{array}$$

In the termination criterion, the same low-dimensional approximation scheme is considered for the computation of the surrogate values of LATE. The MATLAB code for the estimation of LATE is available upon request.

## 3. Simulation Study

The effectiveness of the three causality measures, namely PTE, PTENUE, and LATE, in identifying the true connectivity network of multivariate systems was evaluated in a simulation study, where 100 realizations of each system were considered. The artificial time series were derived from known coupled systems with different characteristics. Additional parameters taken into consideration were the number of variables of the examined system, the time series length, and the coupling strength. The considered time series lengths were n=256,512,1024,2048, and 4096.

For the estimation of PTE, we set the time delay τ=1 (as in the original definition of TE [29]) and the embedding dimension *m* based on each system’s equation, i.e., equal to the maximum delay in the equations of each system. The free parameter $L_{max}$ for the computation of PTENUE and LATE was always fixed slightly larger than *m*. We note that if $L_{max}$ took a sufficiently large value, this did not affect the performance of the measure, while a too large $L_{max}$ would only increase the computational cost of the estimation [33]. The number of nearest neighbors was k=10 (*k* did not affect the performance of the KNN estimator [42]). The number of surrogates for the significance test of PTE and for the stopping criteria of PTENUE and LATE was equal to nsur=100.

For each realization of a multivariate coupled system in *K* variables, there were K(K−1) possible causal influences between the variables. For the identification of the connectivity network of a multivariate system, all possible causal effects were estimated. True positives (TPs) quantify the correct causal influences detected by a causality measure; false positives (FPs) indicate the incorrect link detections; true negatives (TNs) count the correctly identified uncoupled variables; and false negatives (FNs) express the causal links that are spuriously indicated as uncoupled ones by the measure. Eventually, the performance of each causality measure is discussed in terms of its true positive rate:

(6) $$\begin{array}{c} sensitivity=TP/(TP+FN), \end{array}$$

and its true negative rate:

(7) $$\begin{array}{c} specificity=TN/(TN+FP). \end{array}$$

Finally, the F1-score is an indicator of the overall performance of a causality measure:

(8) $$\begin{array}{c} F1-score=2TP/(2TP+FP+FN). \end{array}$$

The empirical findings are discussed in terms of the mean exported sensitivity, specificity, and F1-score of each causality measure over the 100 realizations of each system and each time series length.

### 3.1. Linear Stochastic Process

**Figure 1:**
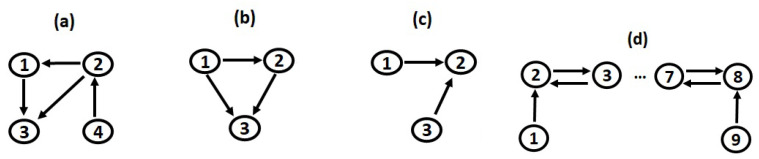
Connectivity network of (**a**) the linear stochastic system in 4 variables, (**b**) the nonlinear stochastic system in 3 variables, (**c**) the chaotic system in 3 variables, and (**d**) the chaotic system in 9 variables.

The first simulation system, denoted as S1, was a linear vector auto-regressive (VAR) model of order five in four variables (Model 1 in [50]):

(9) $$\begin{array}{ccc} x_{1,t} & = & 0.8x_{1,t-1}+0.65x_{2,t-4}+\epsilon_{1,t}\\ x_{2,t} & = & 0.6x_{2,t-1}+0.6x_{4,t-5}+\epsilon_{2,t}\\ x_{3,t} & = & 0.5x_{3,t-3}-0.6x_{1,t-1}+0.4x_{2,t-4}+\epsilon_{3,t}\\ x_{4,t} & = & 1.2x_{4,t-1}-0.7x_{4,t-2}+\epsilon_{4,t} \end{array}$$

where $\epsilon_{i,t}$, i=1,…,4, are Gaussian white noise processes independent of each other with the unit the standard deviation. The true connections of the system are known; $X_{1}\to X_{3}$, $X_{2}\to X_{1}$, $X_{2}\to X_{3}$, and $X_{4}\to X_{2}$ are unidirectional causal relationships (see Figure 1a). Although the three examined causality measures were nonlinear, we considered this simulation system in order to examine their ability in identifying linear causal influences.

**Figure 2:**
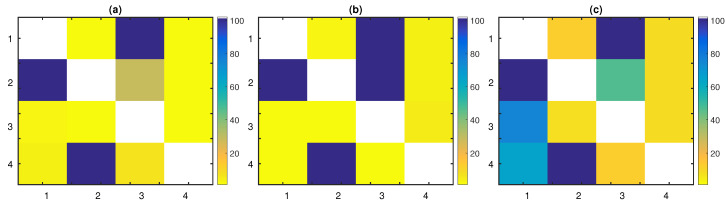
The percentage of significant (**a**) partial transfer entropy (PTE), (**b**) PTE using the non-uniform embedding (NUE) scheme (PTENUE), and (**c**) LATE values, over the 100 realizations of the linear stochastic system with n=1024. The direction of causal influence is from row to column in the matrix representation. The true causal connections are at the matrix elements (1,3), (2,1), (2,3), and (4,2).

PTE had an increasing sensitivity as the the time series length grew, affecting its overall performance. It detected all the true connections with a percentage of 100% over all realizations for all *n*, except for the link $X_{2}\to X_{3}$, for which a large time series length was required in order to achieve a high percentage; varying from 16% for n=256 up to 95% for n=4096. The specificity of PTE was high for all time series lengths, with percentages of the uncoupled directions taking values close to the nominal level (from 0% up to 7%). Since the dimensionality of this system was intermediate, the full conditioning of PTE affected its sensitivity only for small time series lengths. Figure 2a displays the percentage of significant PTE values over the 100 realizations of the linear stochastic process with n=1024.

PTENUE outperformed the other measures, scoring the highest sensitivity, specificity, and subsequently, the highest F1-score for all time series lengths. It captured all the true couplings with a percentage of 100%, but for the link $X_{2}\to X_{3}$; for n=256, the achieved percentage was 49% and for n=512 96%. The corresponding percentages of the uncoupled directions were all close to the nominal level, varying from 0% up to 8%. Empirical findings indicated that partial conditioning did not lead to decreased specificity. On the contrary, PTENUE improved the performance of PTE, achieving higher scores in terms of sensitivity and specificity. In Figure 2b, the percentage of significant PTENUE values over the 100 realizations of the linear stochastic process with n=1024 is presented.

Finally, LATE captured the true causal influences $X_{1}\to X_{3}$, $X_{2}\to X_{1}$, and $X_{4}\to X_{2}$ of the linear stochastic system, with high percentages for all time series lengths (varying from 97% up to 100%). However, the link $X_{2}\to X_{3}$ was detected with increasing percentages with the time series length; we obtained the percentages 43% (n=256), 38% (n=512), 46% (n=1024), 74% (n=2048), and 94% (n=4096), respectively. LATE had the smallest specificity among the three causality measures, which affected its overall performance. The low specificity of LATE was due to the approximation scheme for the computation of the high-dimensional CMI. This approximation also caused the detection of the spurious causal effects $X_{1}\to X_{2}$ (with percentages varying from 12% to 20% for the different time series lengths) and $X_{3}\to X_{1}$ (with percentages varying from 51% to 75% for the different time series lengths). Additional false causal influences were detected by LATE, but with lower percentages and mainly for small *n*, e.g., LATE suggested the link $X_{2}\to X_{4}$ (percentage of detection around 15% for all time series lengths) and $X_{3}\to X_{2}$ (21% for n=256, 16% for n=512). Further, the indirect coupling $X_{4}\to X_{1}$ was revealed with increasing percentages as the time series length grew (from 30% for n=256 up to 83% for n=4096) and $X_{4}\to X_{3}$ (with percentages varying from 6% to 16% for the different time series lengths). The aforementioned findings are summarized in Figure 2c, where this matrix representation of the percentages of significant causal effects over the 100 realizations of the system with n=1024 highlights the fact that LATE captured spurious links and performed poorer than PTE and PTENUE.

The findings regarding the sensitivity, specificity, and F1-score of the three measures, for the linear stochastic system, are revealed in Table 1. The overall performance of each measure was quantified as the mean over all realizations and time series lengths. The mean F1-score for PTE, PTENUE, and LATE was 90.60%, 96.41%, and 76.27%, respectively. Conclusively, the worst performance was obtained by LATE, while PTENUE outperformed the other measures. Most importantly, only LATE gave erroneous causal influences.

### 3.2. Nonlinear Stochastic Process

The second simulation system, denoted as S2, was a nonlinear VAR of order one in three variables (Model 7 in [51]):

(10) $$\begin{array}{ccc} x_{1,t} & = & 3.4x_{1,t-1}\left(1-x_{1,t-1}^{2}\right)e^{-x_{1,t-1}^{2}}+0.4\epsilon_{1,t}\\ x_{2,t} & = & 3.4x_{2,t-1}\left(1-x_{2,t-1}^{2}\right)e^{-x_{2,t-1}^{2}}+0.5x_{1,t-1}x_{2,t-1}+0.4\epsilon_{2,t}\\ x_{3,t} & = & 3.4x_{3,t-1}\left(1-x_{1,t-1}^{3}\right)e^{-x_{1,t-1}^{3}}+0.3x_{2,t-1}+0.5x_{1,t-1}^{2}+0.4\epsilon_{3,t} \end{array}$$

where $\epsilon_{i,t}$, i=1,…,3, are Gaussian white noise processes independent of each other with the unit the standard deviation. The causal link $X_{2}\to X_{3}$ was linear, while $X_{1}\to X_{2}$ and $X_{1}\to X_{3}$ were nonlinear unidirectional ones (see Figure 1b). Therefore, this system incorporated linear and nonlinear causal influences. Additionally, we demonstrated the performance of the examined measures in the case of low-dimensions, where full conditioning was feasible.

**Figure 3:**
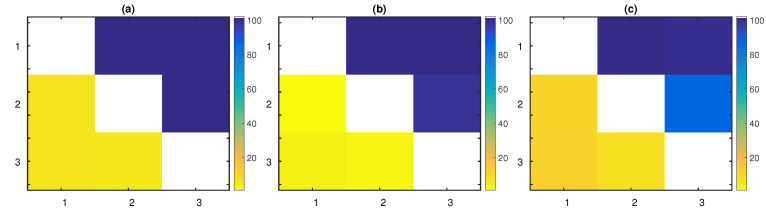
The percentage of the significant (**a**) PTE, (**b**) PTENUE, and (**c**) LATE values, over the 100 realizations of the nonlinear stochastic system with n=512. The direction of causal influence is from row to column in the matrix representation. The true causal connections are at the matrix elements (1,2), (1,3), and (2,3).

As expected, PTE was effective in this low-dimensional system, achieving high sensitivity and specificity, even for small time series lengths. PTENUE also performed well. Although partial conditioning was not required in such a low-dimensional system, PTENUE slightly outperformed PTE, but for n=256. In this case, PTENUE had somewhat smaller sensitivity than PTE, which affected its overall performance. LATE performed also well, but placed again last among the three causality measures. This was due to the slightly lower specificity of LATE compared to that of the other two measures. The percentage of capturing the causal effects $X_{2}\to X_{1}$ and $X_{3}\to X_{1}$ was above the nominal level for LATE; varying from 5% up to 11%. On the other hand, for PTENUE, these percentages stayed very low (between 0% and 3%), while for PTE, they took values from 2% up to 7%. These findings are demonstrated in Figure 3 for n=512.

In conclusion, all the considered measures performed well for low-dimensional systems, achieving high F1-scores, even for small time series lengths (see Table 2). The mean F1-score over all realizations and time series lengths for PTE, PTENUE, and LATE was 97.32%, 97.05%, and 92.82%, respectively. PTE was in first place for n=256, while for larger *n*, PTENUE seemed to be the most effective measure.

### 3.3. Chaotic System: Coupled Hénon Maps

The last simulation system, denoted as S3, consisted of *K* coupled Hénon maps [52]:

$$\begin{array}{ccc} x_{1,t} & = & 1.4-x_{1,t-1}^{2}+0.3x_{1,t-2}\\ x_{i,t} & = & 1.4-0.5cx_{i-1,t-1}x_{i+1,t-1}-\left(1-c\right)x_{i,t-1}^{2}+0.3x_{i,t-2},i=2,\ldots ,K-1.\\ x_{K,t} & = & 1.4-x_{K,t-1}^{2}+0.3x_{K,t-2} \end{array}$$

This is a chaotic system in *K* variables. The parameter *c* controls the coupling strength between the variables, while the performance of the causality measures is examined for increasing values of *K*. In particular, we set K=3, K=9, and K=50.

#### 3.3.1. Coupled Hénon Maps in Three Variables

First, we considered a low-dimensional case and set K=3 variables. Additionally, a moderate coupling strength c=0.3 was assumed. The causal network is presented in Figure 1c; the unidirectional links $X_{1}\to X_{2}$ and $X_{3}\to X_{2}$ existed.

All measures scored high, since a small *K* and a moderate coupling strength were selected (Table 3). PTENUE outranked the other two measures with a mean F1-score over all realizations and time series lengths equal to 99.89%. LATE closely followed, accomplishing a mean F1-score equal to 98.05%. Finally, PTE scored last, achieving a mean F1-score equal to 86.42%. Although PTE captured the direct causal influences, it spuriously indicated additional ones. In particular, the links $X_{2}\to X_{1}$ and $X_{2}\to X_{3}$ were falsely indicated. Thus, PTE had a decreasing specificity with *n*, which influenced its overall outcome.

#### 3.3.2. Coupled Hénon Maps in Nine Variables, Coupling Strength c = 0.3

The performance of the three causality measures was then examined for a higher dimensional case. We set the number of variables equal to K=9, while the coupling strength was fixed to c=0.3. The system was chaotic, while both unidirectional and bidirectional nonlinear causal effects existed (Figure 1d).

**Figure 4:**
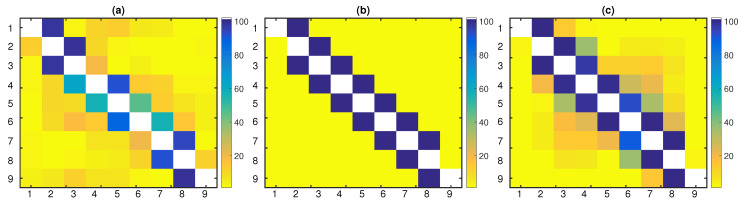
The percentage of significant (**a**) PTE, (**b**) PTENUE, and (**c**) LATE values, over the 100 realizations of the chaotic coupled system in K=9 variables with coupling strength c=0.3 and n=1024. The direction of causal influence is from row to column in the matrix representation.

As the dimensionality of the chaotic system increased, PTE required larger time series lengths to detect the true connections with a high percentage over the 100 realizations. Indirect causal effects were obtained with low, but slightly increasing percentages as the time series length grew. The spurious couplings $X_{2}\to X_{1}$ and $X_{8}\to X_{9}$ also arose as *n* grew. The matrix representation of the extracted significant causal influences based on PTE as a percentage over the 100 realizations of this system with n=1024 is displayed in Figure 4a. The sensitivity of PTE increased from 32% (for n=256) to 99.79% (for n=4096) and affected its overall performance (Table 4). The highest mean F1-score for PTE was achieved for n=2048. Its specificity was high; however, it started to decrease from n=1024.

LATE scored second, accomplishing a mean F1-score equal to 84.48% over all realizations and time series lengths. Its performance was improved as the time series length increased and reached the highest F1-score for n=4096. Specificity was high, however lower than PTENUE’s and PTE’s. LATE indicated some indirect causal influences, such as $X_{2}\to X_{4}$ and $X_{4}\to X_{6}$ (see Figure 4c for n=1024).

PTENUE placed at the top with a very high mean F1-score over all realizations and time series lengths, equal to 98.57%. It was the only causality measure that did not indicate indirect or spurious causal effects (see Figure 4b for n=1024).

#### 3.3.3. Coupled Hénon Maps in Nine Variables, Coupling Strength c=0.1

In order to evaluate the effectiveness of the three causality measures in the case of weak couplings, we considered the chaotic system in K=9 variables, while the coupling strength was fixed to c=0.1. The connectivity pattern was the same (as in Figure 1d). The considered time series length for this case was n=1024 and 2048.

PTE seemed to be completely ineffective in the case of weak couplings (Table 5). On the other hand, LATE and PTENUE achieved a high mean F1-score. For n=1024, LATE outperformed PTENUE, while the opposite was the case for n=2048. Both measures were able to detect the true causal effects, despite the weak coupling strength. As *n* increased, the extracted scores of both measures improved. Overall, the optimal performance was by LATE, with a mean F1-score of 92.41%, while PTENUE closely followed with a mean F1-score equal to 92.21%.

#### 3.3.4. Coupled Hénon Maps in K=50 Variables

Finally, we set K=50 variables and kept the coupling strength fixed to c=0.3. Since the number of variables was pretty large, we refrained from making calculations for all time series lengths and extracted results only for n=2048. To reduce the computational cost, the number of realizations was also limited to 10.

PTE failed to capture the connectivity network of this high-dimensional system (Table 6). On the other hand, results indicated the efficiency of PTENUE to capture only the true causal influences, achieving a high sensitivity, specificity, and eventually, F1-score. LATE detected the true couplings, but with lower percentages over all the realizations compared to PTENUE. The percentages of significant LATE values for the true connections varied from 40% to 100% over the 10 realization. Further, LATE indicated some indirect and spurious links, e.g., the spurious link $X_{2}\to X_{1}$ was obtained with a percentage equal to 20%.

Based on the empirical findings, the performance of PTE substantially deteriorated as the dimension of the examined system increased, while seeming to be ineffective in the case of weak couplings. The full conditioning in PTE resulted in a decreased sensitivity in the case of high dimensions. Additionally, PTE may indicate spurious couplings. On the other hand, PTENUE outperformed the other measures in the majority of the examined cases. A sufficient time series length was required to achieve its optimal performance. Partial conditioning was performed taking into consideration the higher order informational contributions, and redundant lagged terms were excluded from the mixed embedding vector. This way, dimension reduction was efficiently performed, and PTENUE effectively faced the “curse of the dimensionality”. LATE performed satisfactorily for the majority of the simulated systems. It outperformed the other measures in the case of weak coupling and small time series length. However, LATE achieved in general lower sensitivity and specificity compared to PTENUE, while indicating spurious couplings, due to the CMI approximation.

To summarize the above results, all measures performed satisfactorily in the case of low-dimensional systems and sufficiently large time series lengths. The exported mean F1-scores over all realizations, time series lengths, and simulation systems for PTE, PTENUE, and LATE were 63.37%, 96.73%, and 83.86%, respectively. PTENUE prevailed over the other two measures; it correctly identified the causal influences of both low- and high-dimensional multivariate systems, accomplishing high scores in all examined cases.

### 3.4. Computational Cost

The computational cost of the three measures was also essential, especially in the case of high-dimensional systems. PTENUE had the least computational cost due to its dimension reduction computational scheme. As the dimensionality and the time series length increased, its computational superiority over the other two measures was even more pronounced. In the case of low-dimensional systems, LATE seemed to be the most burdensome one; the estimation of many low-dimensional CMI terms was more demanding than the estimation of a higher dimensional CMI. Raising the time series length and the dimensionality, PTE became the most demanding measure, since full conditioning was performed.

**Figure 5:**
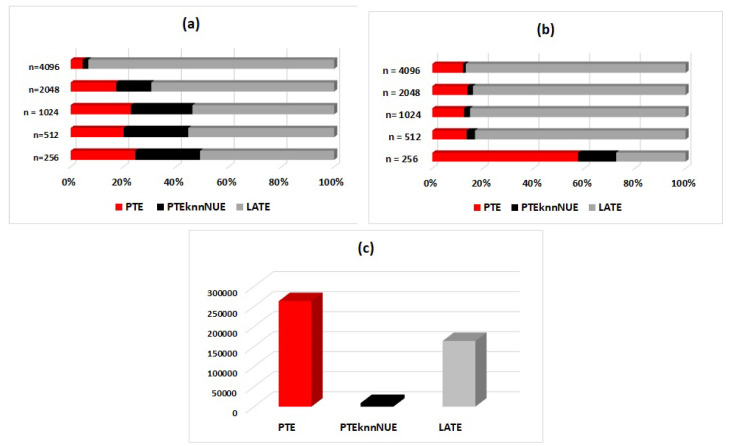
The computational cost (in seconds) of the causality measures PTE, PTENUE, and LATE displayed by 3D 100% stacked bars of (**a**) the nonlinear stochastic system (S2) in 3 variables, (**b**) the chaotic system (S3) in 9 variables, and (**c**) the chaotic system (S3) in K=50 variables.

To better demonstrate the corresponding findings from the analysis, we also display the seconds required by each measure in order to complete the estimations for one realization of a multivariate system, i.e., for identifying its connectivity network. Outcomes were observed in regards to the time series lengths. Results are indicatively displayed in Figure 5 for S2 in K=3 variables (a low-dimensional system), for S3 in K=9 variables (intermediate dimensionality), and for S3 in K=50 variables (a high-dimensional system).

The specifications of the computer used to run the simulations are as follows. MATLAB version: R2015a; Operating system: Windows 10 Pro (64 bit); Processor: Intel(R) Core(TM) i5-3470CPU @ 3.20GHz; RAM: 4GB.

## 4. Application

In this section, we demonstrate the applicability of the three causality measures to financial time series. In particular, we examine the connectivity pattern between stocks of index CAC40. The stock market index CAC40 is a reference point for the French stock market Euronext (ex Paris Bourse) and consists of the weighted capitalization of the 40 companies (among the 100 highest market caps) with the highest capitalization traded in this stock market. The companies that form the CAC40 index can be reviewed at https://en.wikipedia.org/wiki/CAC_40.

The examined time period was 2001-12-14 to 2018-09-04. The composition of the index was quarterly reviewed. The number of the observations was n=4303. Two variables were excluded from the analysis due to too many missing observations. Therefore, the number of variables of the analysis was K=38. To achieve stationarity, analysis was performed based on the logarithmic returns. All series were obtained from Yahoo finance.

For the calculations, the embedding dimension for the computation of PTE was m=2, and the delay was τ=1. We set the number of neighbors k=10, the number of surrogates nsur=100, the significance level for PTENUE α=0.01, while for PTE, α=0.05. Finally, we fixed $L_{max}=5$ for the estimation of PTENUE and LATE.

**Figure 6:**
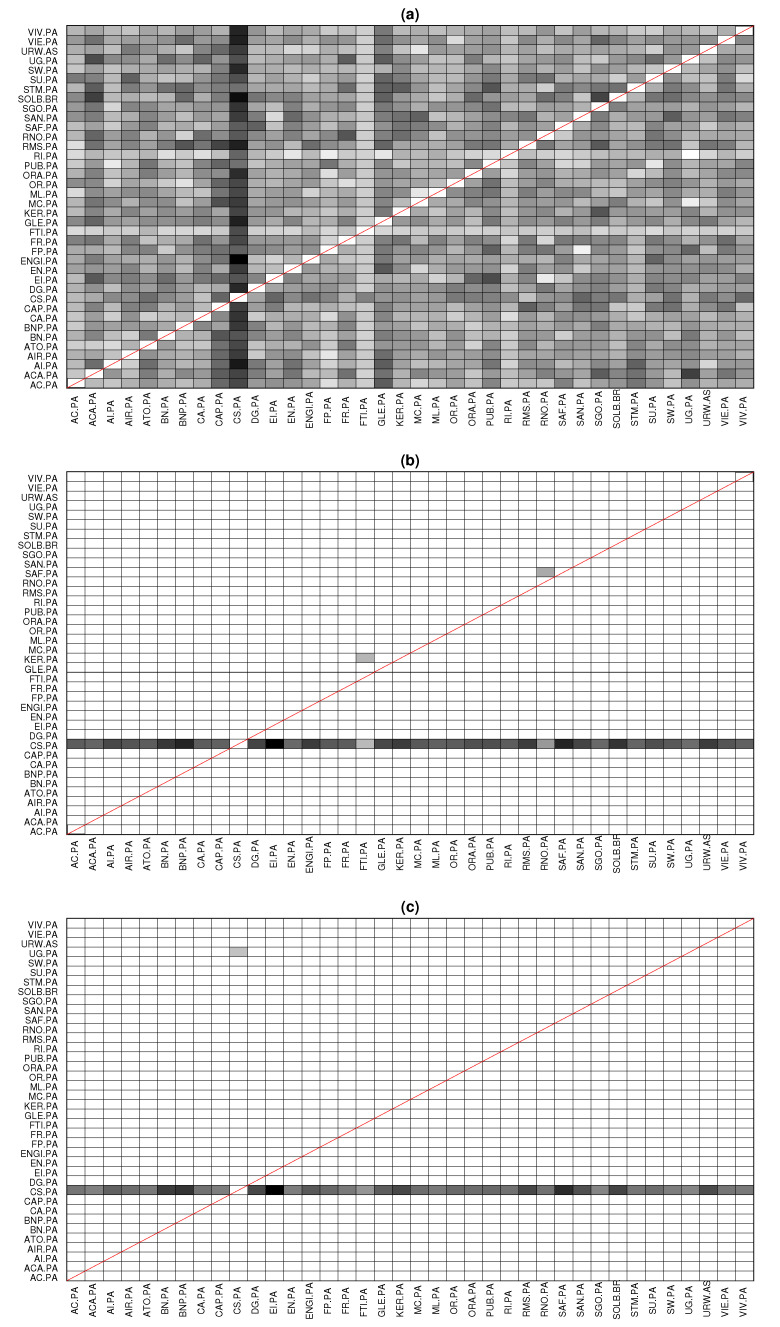
Extracted causal links of CAC40 based on (**a**) PTE, (**b**) PTENUE, and (**c**) LATE. Rows drive the columns. The diagonal of this matrix representation is marked in red.

The goal of this application was to determine the leading forces in CAC40, i.e., we were seeking to find the most significant driving variables. PTE identified all couplings as significant, and therefore, the results suggested an almost fully connected network. On the other hand, PTENUE and LATE determined the most significant leading variable to be CS.PA (AXA SA, a French multinational insurance firm), influencing all the other observed variables. Although PTENUE and LATE indicated the same leading driving variable, few different additional links were obtained. Specifically, PTENUE indicated two additional links: FTI.PA → KER.PA, where FTI.PA represents in the stock market TechnipFMC (a Franco-American oil services firm) and KER.PA represents KERING S.A. (a world-class luxury group that develops, designs, manufactures, markets, and sells apparel and accessories), and RNO.PA → SAF.PA, where RNO.PA represents Renault S.A. (a vehicle manufacturing and distribution company) and SAF.PA represents Safran S.A. (a company that engages in the design, manufacture, and sale of aircraft, defense, and communication equipment and technologies). On the other hand, LATE suggested the coupling UG.PA → CS.PA, where UG.PA represents Peugeot (a French car manufacturer firm and part of Groupe PSA). The connectivity pattern obtained by the three causality measures is displayed in Figure 6.

We note that consistent results were identified using the partial mutual information on mixed embedding (PMIME) [33] in [53], when analyzing the same dataset. In particular, CS.PA was identified as the most significant driving variable by observing the extracted connectivity network and calculating the in- and out-degree of the variables.

The most common analyses for constructing financial networks were based on the linear Granger causality [18,54], e.g., [55,56,57], and on correlation analysis [58,59,60]. For comparative reasons, we also report the exported results based on standard linear approaches. Utilizing the conditional Granger causality index, 242 causal links were obtained if the order of the VAR was equal to P=1 (as suggested by the Bayesian information criterion [61]), while 348 links were obtained for P=2 (as suggested by the Akaike information criterion [62]). Based on the partial correlation coefficient, six-hundred ten links were detected. Thus, both methods seemed to overestimate the number of connections in the examined financial network.

## 5. Conclusions

In this paper, three conditional causality measures were briefly reviewed and evaluated on artificial datasets. In particular, we examined the performance of partial transfer entropy (PTE) computed based on the standard uniform embedding scheme, PTE estimated using the non-uniform embedding (NUE) scheme, denoted as PTENUE, and PTE using the NUE scheme and also a low-dimensional approximation (LA) method for the computation of conditional mutual information (CMI), denoted as LATE. We assessed the effectiveness of each causality measure in identifying the causal influences among the variables of complex coupled systems based on the extracted sensitivity, specificity, and F1-score. In the simulation analysis, we controlled the dimension of the data, the sample size, the coupling strength, and its nature (linear or nonlinear, unidirectional, or bidirectional). The dimensionality of the chaotic simulation system advanced up to 50 variables in order to reflect the complexity of the real data. Finally, the applicability of the three causality measures in real applications was demonstrated using financial time series.

Based on the empirical findings, partial transfer entropy (PTE) was mainly efficient in the case of low-dimensional systems. LATE improved the performance of PTE in the case of high-dimensional coupled systems. LATE had an overall good performance and was particularly effective in the case of weak couplings and small time series lengths. However, it was computationally expensive and may indicate spurious causal influences.

We should note here that the simulation results displayed in [35] substantially deviated from the outcomes of this study regarding the performance of LATE; however, our results seemed to be in agreement with the reported results in [36], where also low-dimensional approximation methods for CMI were employed.

PTENUE outperformed the other measures by accurately identifying the connectivity network of all the examined scenarios and achieving the highest mean F1-score over all the examined scenarios. As the dimension of the examined systems progressively increased, the necessity of using dimension reduction techniques, such as the NUE scheme, was profound. Novel simulation results were presented for both low- and high-dimensional systems, where the number of variables varied from K=3 up to K=50. PTENUE did not seem to be significantly affected by the “curse of dimensionality”, while remaining effective also in case of weakly coupled variables. Additionally, PTENUE was the least computationally demanding measure among the three examined measures.

In the financial applications, the connectivity network of CAC40 was examined in order to determine the leading forces. Both PTENUE and LATE indicated the most significant leading variable to be CS.PA (AXA SA, a French multinational insurance firm), influencing all the other observed variables. PTE failed to identify the connectivity relationships of CAC40, tending to identify a fully coupled network.

Subsequently, PTENUE was the most effective causality measure for identifying the connectivity network of multivariate systems among the three examined measures. It was robust, and it was unaffected by the “curse of dimensionality” and the nature, and the strength of the connections. However, the sample size should be sufficiently large, and this should be jointly considered with the dimensionality of the examined data. Finally, it had the least computational cost.

The presented outcomes concerned multivariate systems with a sparse causality structure. The examined causality measures were probabilistic approaches that required the stationarity of the time series and could be applied only to time-invariant networks. Additionally, contemporaneous relationships were not considered. Future work involves the identification of the efficiency of PTENUE on multivariate systems of higher dimensions and with denser causality structures, which will be utilized in financial applications, such as for portfolio construction. A forthcoming work will examine the robustness of PTENUE in the case of noisy data, where preliminary results seemed very promising.

## Figures and Tables

**Table 1:** Sensitivity, specificity, and F1-score obtained from 100 realizations of the linear stochastic process.

PTE	Sensitivity	Specificity	F1-Score
n = 256	79	97.75	86.07
n = 512	79	97.75	86.19
n = 1024	82	98.62	88.52
n = 2048	91.5	98.88	94.15
n = 4096	98.75	98.62	98.09
**PTENUE**	**Sensitivity**	**Specificity**	**F1-Score**
n = 256	87.25	97	90.21
n = 512	99	97.38	97.13
n = 1024	100	98.38	98.64
n = 2048	100	97.75	98.04
n = 4096	100	97.75	98.04
**LATE**	**Sensitivity**	**Specificity**	**F1-Score**
n = 256	85	80	76.22
n = 512	84.5	75	72.96
n = 1024	86.5	75.75	74.18
n = 2048	93.5	73	76.01
n = 4096	98.5	78	81.99

**Table 2:** Sensitivity, specificity, and F1-score obtained from 100 realizations of the nonlinear stochastic process.

PTE	Sensitivity	Specificity	F1-Score
n = 256	94	95	94.2
n = 512	100	94.33	97.7
n = 1024	100	95.67	98.18
n = 2048	100	97.33	98.86
n = 4096	100	94.33	97.68
**PTENUE**	**Sensitivity**	**Specificity**	**F1-Score**
n = 256	83.33	99.67	89.07
n = 512	99.33	98	98.74
n = 1024	100	98.33	99.29
n = 2048	100	97.67	99
n = 4096	100	98	99.14
**LATE**	**Sensitivity**	**Specificity**	**F1-Score**
n = 256	73.33	93	79.9
n = 512	95	90.67	93.21
n = 1024	99.67	92.67	96.66
n = 2048	100	94.33	97.57
n = 4096	100	92.33	96.75

**Table 3:** Sensitivity, specificity, and F1-score obtained from 100 realizations of the chaotic system in K=3 variables with c=0.3.

PTE	Sensitivity	Specificity	F1-Score
n = 256	99.5	90	92
n = 512	100	86.75	90.3
n = 1024	100	82.75	87.79
n = 2048	100	74.5	82.19
n = 4096	100	70.5	79.8
**PTENUE**	**Sensitivity**	**Specificity**	**F1-Score**
n = 256	100	99.25	99.47
n = 512	100	100	100
n = 1024	100	100	100
n = 2048	100	100	100
n = 4096	100	100	100
**LATE**	**Sensitivity**	**Specificity**	**F1-Score**
n = 256	96.65	96.75	94.47
n = 512	100	96.25	97
n = 1024	100	98.5	98.8
n = 2048	100	100	100
n = 4096	100	100	100

**Table 4:** Sensitivity, specificity, and F1-score obtained from 100 realizations of the chaotic system in K=9 variables with coupling strength c=0.3.

PTE	Sensitivity	Specificity	F1-Score
n = 256	32	95.17	40.76
n = 512	50.57	95.72	59.3
n = 1024	72.21	95.16	75.23
n = 2048	89.43	92.22	81.12
n = 4096	99.79	81.67	72.83
**PTENUE**	**Sensitivity**	**Specificity**	**F1-Score**
n = 256	92.71	98.91	93.91
n = 512	99.57	99.57	98.92
n = 1024	100	100	100
n = 2048	100	100	100
n = 4096	100	100	100
**LATE**	**Sensitivity**	**Specificity**	**F1-Score**
n = 256	72	93.03	70.06
n = 512	90.29	90.95	79.37
n = 1024	98.07	92.41	85.76
n = 2048	100	95.05	91.08
n = 4096	100	97.97	96.11

**Table 5:** Sensitivity, specificity, and F1-score obtained from 100 realizations of the chaotic system in K=9 variables with coupling strength c=0.1.

PTE	Sensitivity	Specificity	F1-Score
n = 1024	5.71	94.52	8.47
n = 2048	4.21	95.19	6.19
**PTENUE**	**Sensitivity**	**Specificity**	**F1-Score**
n = 1024	73.57	100	84.42
n = 2048	100	100	100
**LATE**	**Sensitivity**	**Specificity**	**F1-Score**
n = 1024	79.93	99.29	87.02
n = 2048	96.93	99.71	97.79

**Table 6:** Sensitivity, specificity, and F1-score obtained from 10 realizations of the coupled Hénon maps in K=50 variables for coupling strength c=0.3 and time series length n=2048.

Measure	Sensitivity	Specificity	F1-Score
**PTE**	32.29	97.33	32.69
**PTENUE**	99.17	99.71	96.24
**LATE**	78.85	97.96	67.66

## References

[1] Reinchenbach H. (1978). The Causal Structure of the World and the Difference between Past and Future. Hans Reichenbach Seected Writings 1909–1953: Volume Two.

[2] Reichenbach H. (1956). The Direction of Time.

[3] Wu M.H., Frye R., Zouridakis G. (2011). A comparison of multivariate causality based measures of effective connectivity. Comput. Biol. Med..

[4] Papana A., Kyrtsou C., Kugiumtzis D., Diks C. (2013). Simulation study of direct causality measures in multivariate time series. Entropy.

[5] Siggiridou E., Koutlis C., Tsimpiris A., Kimiskidis V., Kugiumtzis D. Causality networks from multivariate time series and application to epilepsy. Proceedings of the 37th Annual International Conference of the IEEE, Engineering in Medicine and Biology Society (EMBC).

[6] Franciotti R., Falasca N. (2018). The reliability of conditional Granger causality analysis in the time domain. PeerJ Prepr..

[7] Krakovská A., Jakubík J., Chvosteková M., Coufal D., Jajcay N., Paluš M. (2018). Comparison of six methods for the detection of causality in a bivariate time series. Phys. Rev. E.

[8] Shen X., Ma S., Vemuri P., Simon G. (2020). Challenges and opportunities with causal Discovery Algorithms: Application to Alzheimer’s pathophysiology. Sci. Rep..

[9] Eberhardt F. (2017). Introduction to the foundations of causal discovery. Int. J. Data Sci. Anal..

[10] Good I. (1961). A causal calculus (I). Br. J. Philos. Sci..

[11] Suppes P. (1970). A Probabilistic Theory of Causality.

[12] Spirtes P., Glymour C., Scheines R., Kauffman S., Aimale V., Wimberly F. Constructing Bayesian Network Models of Gene Expression Networks from Microarray Data. https://pdfs.semanticscholar.org/f55f/a68d26da6850b43bd9eb10410ca739be1bce.pdf.

[13] Glymour C., Zhang K., Spirtes P. (2019). Review of causal discovery methods based on graphical models. Front. Genet..

[14] Hutter M. (2007). Algorithmic information theory: A brief non-technical guide to the field. arXiv.

[15] Janzing D., Steudel B. (2010). Justifying Additive Noise Model-Based Causal Discovery via Algorithmic Information Theory. Open Syst. Inf. Dyn..

[16] Zenil H., Kiani N., Marabita F., Deng Y., Elias S., Schmidt A., Ball G., Tegnér J. (2019). An algorithmic information calculus for causal discovery and reprogramming systems. iScience.

[17] Wiener N., Beckenbach E. (1956). The theory of prediction. Modern Mathematics for Engineers.

[18] Granger C.W.J. (1969). Investigating causal relations by econometric models and cross-spectral methods. Econometrica.

[19] Cartwright N. (2010). Hunting Causes and Using Them: Approaches in Philosophy and Economics.

[20] James R., Crutchfield J. (2017). Multivariate dependence beyond Shannon information. Entropy.

[21] Eichler M. (2013). Causal inference with multiple time series: Principles and problems. Philos. Trans. R. Soc. A.

[22] Kantz H., Schreiber T. (2004). Nonlinear Time Series Analysis.

[23] Small M. (2005). Applied Nonlinear Time Series Analysis: Applications in Physics, Physiology and Finance.

[24] Boccaletti S., Latora V., Moreno Y., Chavez M., Hwang D.U. (2006). Complex networks: Structure and dynamics. Phys. Rep..

[25] Newman M. (2018). Networks.

[26] Chartrand G. (2006). Introduction to Graph Theory.

[27] Blinowska K., Kuś R., Kamiński M. (2004). Granger causality and information flow in multivariate processes. Phys. Rev. E.

[28] Granger C. (1980). Testing for causality: A personal viewpoint. J. Econ. Dyn. Control.

[29] Schreiber T. (2000). Measuring information transfer. Phys. Rev. Lett..

[30] Papana A., Kugiumtzis D., Larsson P. (2012). Detection of direct causal effects and application to epileptic electroencephalogram analysis. Int. J. Bifurc. Chaos.

[31] Vlachos I., Kugiumtzis D. (2010). Nonuniform state-space reconstruction and coupling detection. Phys. Rev. E.

[32] Faes L., Nollo G., Porta A. (2011). Information-based detection of nonlinear Granger causality in multivariate processes via a nonuniform embedding technique. Phys. Rev. E.

[33] Kugiumtzis D. (2013). Direct-coupling information measure from nonuniform embedding. Phys. Rev. E.

[34] Montalto A., Faes L., Marinazzo D. (2014). MuTE: A MATLAB toolbox to compare established and novel estimators of the multivariate transfer entropy. PloS ONE.

[35] Zhang J. (2018). Low-dimensional approximation searching strategy for transfer entropy from non-uniform embedding. PLoS ONE.

[36] Jia Z., Lin Y., Liu Y., Jiao Z., Ma Y., Wang J. (2019). Detecting causality in multivariate time series via non-uniform embedding. Entropy.

[37] Verdes P. (2005). Assessing causality from multivariate time series. Phys. Rev. E.

[38] Vakorin V., Krakovska O., McIntosh A. (2009). Confounding effects of indirect connections on causality estimation. J. Neurosci. Methods.

[39] Dorval A. (2008). Probability distributions of the logarithm of inter-spike intervals yield accurate entropy estimates from small datasets. J. Neurosci. Methods.

[40] Raykar V. Probability Density Function Estimation by Different Methods. https://pdfs.semanticscholar.org/e42b/6c1d3165b4e13c3f0ed0fc6c6a26fe029468.pdf.

[41] Sricharan K., Raich R., Hero A. Boundary compensated k-NN graphs. Proceedings of the IEEE International Workshop on Machine Learning for Signal Processing.

[42] Kraskov A., Stögbauer H., Grassberger P. (2004). An introduction to variable and feature selection. Phys. Rev. E.

[43] Papana A., Kugiumtzis D. (2009). Evaluation of mutual information estimators for time series. Int. J. Bifurc. Chaos.

[44] Quiroga R., Kraskov A., Kreuz T., Grassberger P. (2002). Performance of different synchronization measures in real data: A case study on electroencephalographic signals. Phys. Rev. E.

[45] Yu G.H., Huang C.C. (2001). A distribution free plotting position. Stoch. Environ. Res. Risk Assess..

[46] Battiti R. (1994). Using mutual information for selecting features in supervised neural net learning. IEEE Trans. Neural Netw..

[47] Yang H., Moody J. (2000). Data visualization and feature selection: New algorithms for nongaussian data. Advances in Neural Information Processing Systems.

[48] Fleuret F. (2004). Fast binary feature selection with conditional mutual information. J. Mach. Learn. Res..

[49] Guyon I., Elisseeff A. (2003). An introduction to variable and feature selection. J. Mach. Learn. Res..

[50] Winterhalder M., Schelter B., Hesse W., Schwab K., Leistritz L., Klan D., Bauer R., Timmer J., Witte H. (2005). Comparison of linear signal processing techniques to infer directed interactions in multivariate neural systems. Signal Process..

[51] Gourévitch B., Le Bouquin-Jeannès R., Faucon G. (2006). Linear and nonlinear causality between signals: Methods, examples and neurophysiological applications. Biol. Cybern..

[52] Politi A., Torcini A. (1992). Periodic orbits in coupled Hénon maps: Lyapunov and multifractal analysis. Chaos.

[53] Kyrtsou C., Mikropoulou C., Papana A. Addressing the “curse of dimensionality” when testing for Granger causality. Application to a set of financial assets. Proceedings of the Conference on Complex Networks.

[54] Geweke J. (1982). Measurement of linear dependence and feedback between multiple time series. J. Am. Stat. Assoc..

[55] Billio M., Getmansky M., Lo A., Pelizzon L. (2012). Econometric measures of connectedness and systemic risk in the finance and insurance sectors. J. Financ. Econ..

[56] Yao C.Z., Lin J.N., Lin Q.W., Zheng X.Z., Liu X.F. (2016). A study of causality structure and dynamics in industrial electricity consumption based on Granger network. Physica A.

[57] Gao X., Huang S., Sun X., Hao X., An F. (2018). Modelling cointegration and Granger causality network to detect long-term equilibrium and diffusion paths in the financial system. R. Soc. Open Sci..

[58] Mantegna R. (1999). Hierarchical structure in financial markets. Eur. Phys. J. B.

[59] Chi K., Liu J., Lau F. (2010). A network perspective of the stock market. J. Empir. Financ..

[60] Millington T., Niranjan M. (2020). Partial correlation financial networks. Appl. Netw. Sci..

[61] Schwarz G. (1978). Estimating the dimension of a model. Ann. Stat..

[62] Akaikei H. Information theory and an extension of maximum likelihood principle. Proceedings of the 2nd International Symposium on Information Theory.

